# Case of reversible Mobitz type II atrioventricular block after the use of injectable antipsychotics

**DOI:** 10.1002/ccr3.5326

**Published:** 2022-01-27

**Authors:** Hisao Naono, Ryuichiro Takeda, Hiroyuki Masuyama, Jiro Kawano, Keiko Naono‐Nagatomo, Yasushi Ishida

**Affiliations:** ^1^ Social Medical Corporation Doshinkai Koga General Hospital Miyazaki City Japan; ^2^ Department of Medical Psychiatry Miyazaki Prefectural Hospital Miyazaki City Japan; ^3^ 12952 Department of Health Care and Safety Center University of Miyazaki Miyazaki City Japan; ^4^ Department of Medical Cardiology Miyazaki Prefectural Hospital Miyazaki City Japan; ^5^ Department of Health and Welfare Miyazaki Prefectural Mental Health Welfare Center Miyazaki City Japan; ^6^ 12952 Department of Psychiatry Division of Clinical Neuroscience Faculty of Medicine University of Miyazaki Miyazaki City Japan

**Keywords:** haloperidol, levomepromazine, Mobitz II type atrioventricular block, Schizophrenia, sudden cardiac death

## Abstract

Although Mobitz type II atrioventricular block is typically an arrhythmia arising from a permanent organic disorder of the His‐Purkinje system, reversible factors should also be considered. Here, we report the association between a rare reversible Mobitz type II atrioventricular block and antipsychotic medication in a 75‐year‐old patient with schizophrenia.

## Graphical abstract

Mobitz type II atrioventricular blocks may be detected in schizophrenia patients receiving increased doses of antipsychotics on electrocardiogram. Antipsychotics dosage should be more conservative in the elderly.

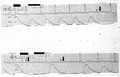

## 1 INTRODUCTION

The risk of drug‐induced arrhythmia and sudden cardiac death associated with the use of antipsychotic drugs has been reported previously.^1^, ^2^ Drug‐induced QT prolongation syndrome is the most common secondary QT prolongation syndrome; its causative agents include antiarrhythmics, antiallergics, antivirals, antitumor drugs, antibiotics, and antipsychotics.^3^ Mobitz type II atrioventricular (AV) block, an arrhythmia caused by a permanent organic disorder of the His‐Purkinje system, predisposes patients to Adams–Stokes attacks and complete AV block.^4^ Although Mobitz II type AV block is typically an arrhythmia arising from a permanent organic disorder of the His‐Purkinje system, reversible factors should also be considered. However, reports describing antipsychotic‐induced Mobitz type II AV block are limited. Here, we present a case of reversible Mobitz II type AV block due to a sudden increase in agitation after receiving intramuscular injections of typical antipsychotics (haloperidol and levomepromazine) in a patient with schizophrenia. Informed consent was obtained from the patient and his family for the publication of this report.

## 2 CASE HISTORY

A 75‐year‐old man with stage II schizophrenia had been receiving treatment for approximately 50 years since disease onset. He had no history of palpitations, dizziness, or fainting spells. At the end of March 2018, he was admitted to the psychiatric department of another hospital. His delusional state with auditory hallucinations and psychomotor agitation worsened, necessitating daily intramuscular injections of haloperidol and levomepromazine. The total haloperidol and levomepromazine dosages were 25 mg/5 days and 100 mg/4 days, respectively. After the final injection, the patient gradually experienced difficulty moving and experienced sudden‐onset pallor and severe pulselessness and perspiration, following which he went into a state of shock and was transferred to our hospital.

On admission, the patient was stuporous (Glasgow Coma Scale score: E2V2M2). His body temperature was 34.8°C, his heart rate was 45/min, and his blood pressure was 60/40 mm Hg. Spontaneous breathing was weak, but the light reflex was normal. Neurological examination revealed no upper or lower limb paralysis, bilateral elbow joint contracture in flexion due to extrapyramidal symptoms, neck retroflexion, and tardive oral dyskinesia. The drug‐induced extrapyramidal symptoms scale^5^ score was 29. Hematological and biochemical test results indicated a red blood cell count of 340 × 10^4^/uL, hemoglobin level of 10.6 g/dl, blood urea nitrogen level of 10.7 mg/dl, creatinine concentration of 0.59 mg/dl, Na of 138mEq/L, K of 3.34 mEq/L, Cl of 101.0 mEq/L, Mg of 1.4 mg/dl, glycated hemoglobin of 6.9%, thyroid‐stimulating hormone level of 2.6 µU/ml, FT4 of 1.02ng/dl, troponin negativity, and a creatine kinase‐myocardial band level of 15 IU/L. Arterial blood gas and blood coagulation system test results were normal. Head computed tomography revealed no acute lesions. Electrocardiography (ECG) revealed an HR of 48 beats/min; no axial deviation; complete right bundle branch block; I‐degree AV block; a negative, flat, low, biphasic T wave in I, II, aVR, aVL, aVF, and V1‐6; and a QTc of 480 ms (Bazett correction). Echocardiography in the emergency room revealed normal motion, 57% left ventricular ejection fraction, no mitral regurgitation, no echo‐free space, and no intimal flap.

## 3 DIFFERENTIAL DIAGNOSIS, INVESTIGATIONS, AND TREATMENT

**FIGURE 1:**
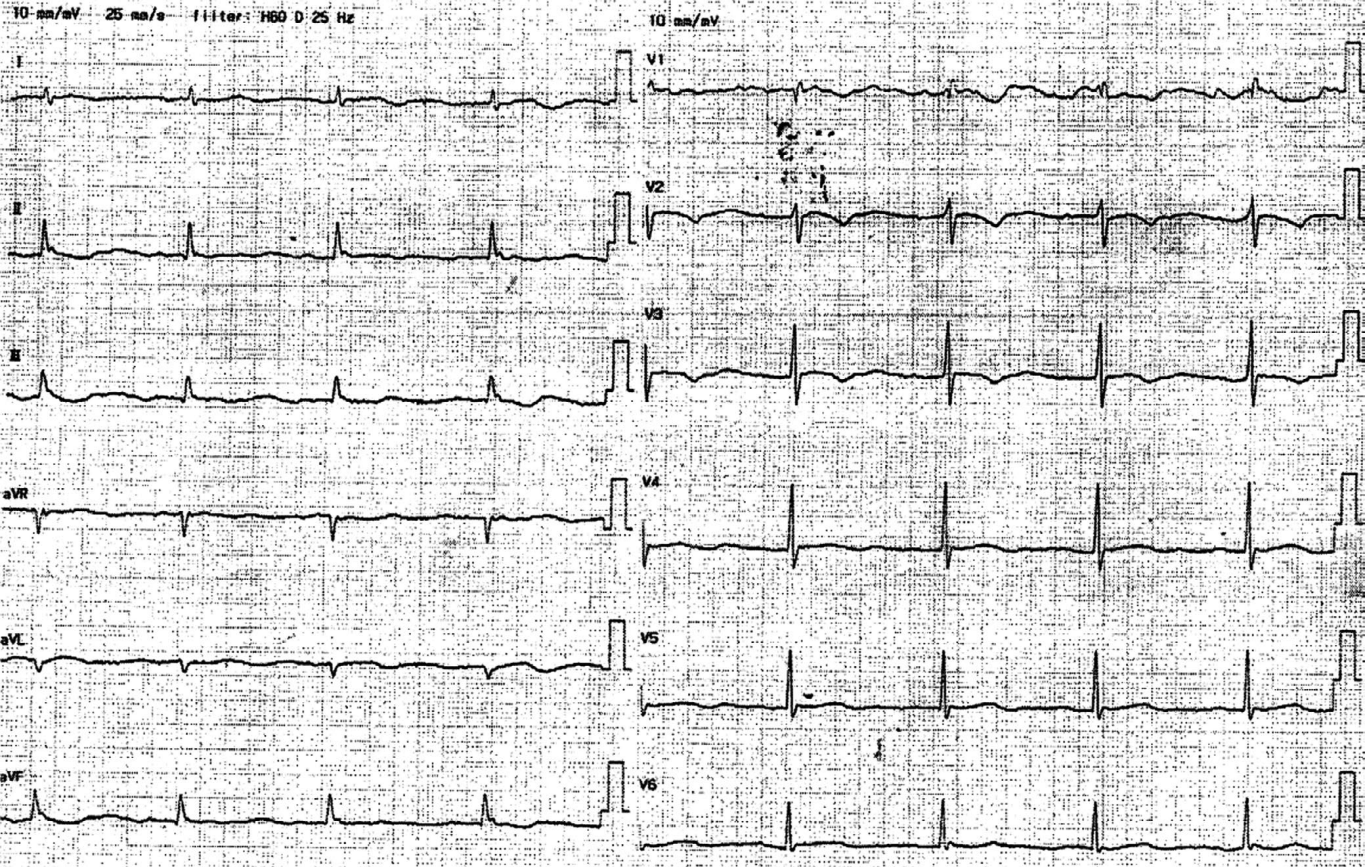
ECG on admission shows a HR of 48 beats/min, no axial deviation, complete right bundle branch block, I‐degree AV block I, II, aVR, aVL, aVF, V1‐6 negative T waves, flat and low T waves, biphasic changes, and a QTc of 480 ms (Bazett correction)

**FIGURE 2:**
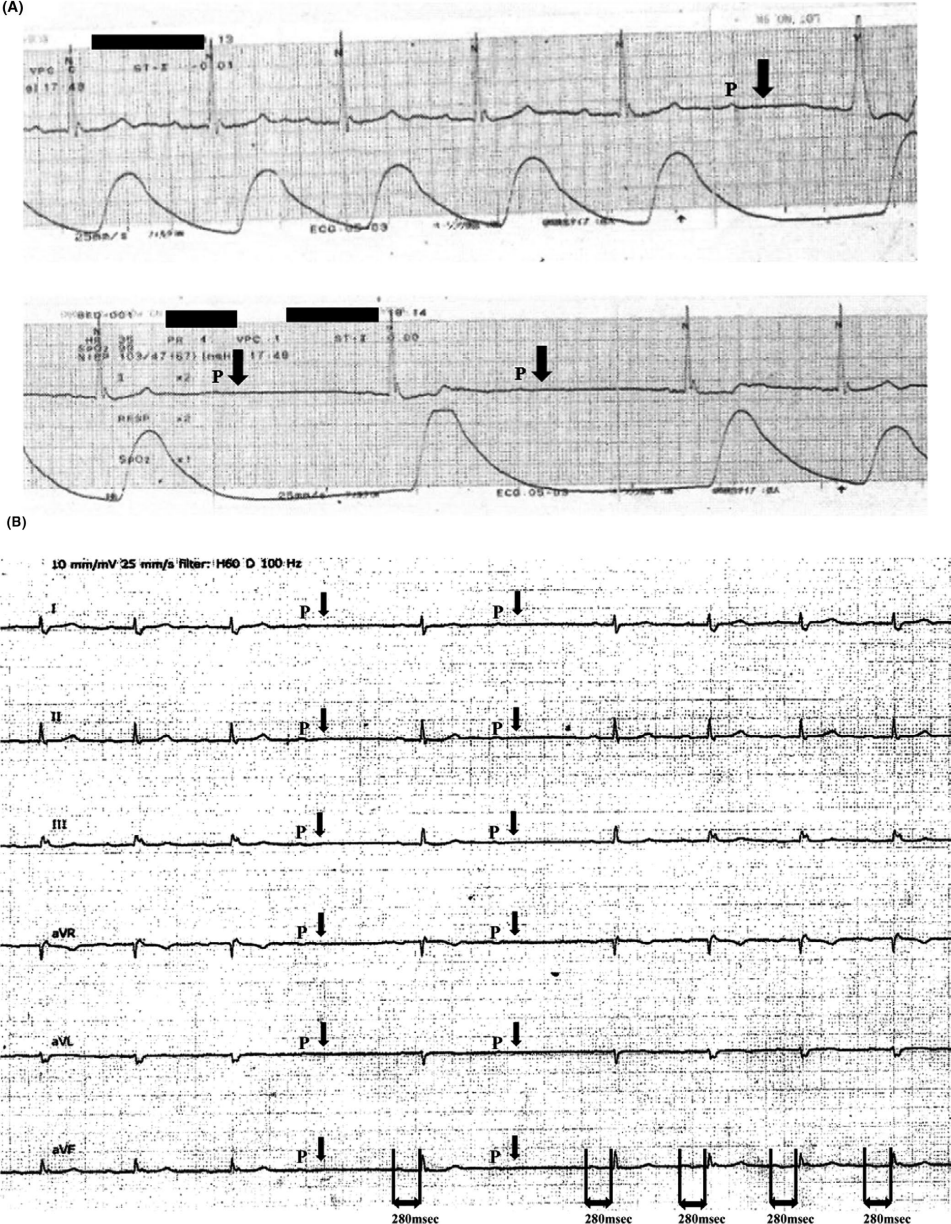
(A) Electrocardiogram monitoring detected QRS wave loss. Arrows indicate dropped QRS wave. (B) ECG on admission to the ICU showing Mobitz type II atrioventricular block with QRS wave loss and RR intervals of up to less than 2 s. The PR interval in the conducted beats remained constant (PR=280 msec). “P” indicates P wave

Based on our findings, the patient was diagnosed with bradyarrhythmia, QT prolongation, hypomagnesemia, and schizophrenia (Figure 1). After admission, all antipsychotics were discontinued, and the QT prolongation improved after correction for low magnesium levels; however, the bradycardia persisted. During this period, a Mobitz type II AV block with QRS wave loss and RR intervals of up to 2 s appeared (Figure 2a,b). The patient was transferred to the intensive care unit (ICU) for respiratory and circulatory management.

## 4 OUTCOME AND FOLLOW‐UP

**FIGURE 3:**
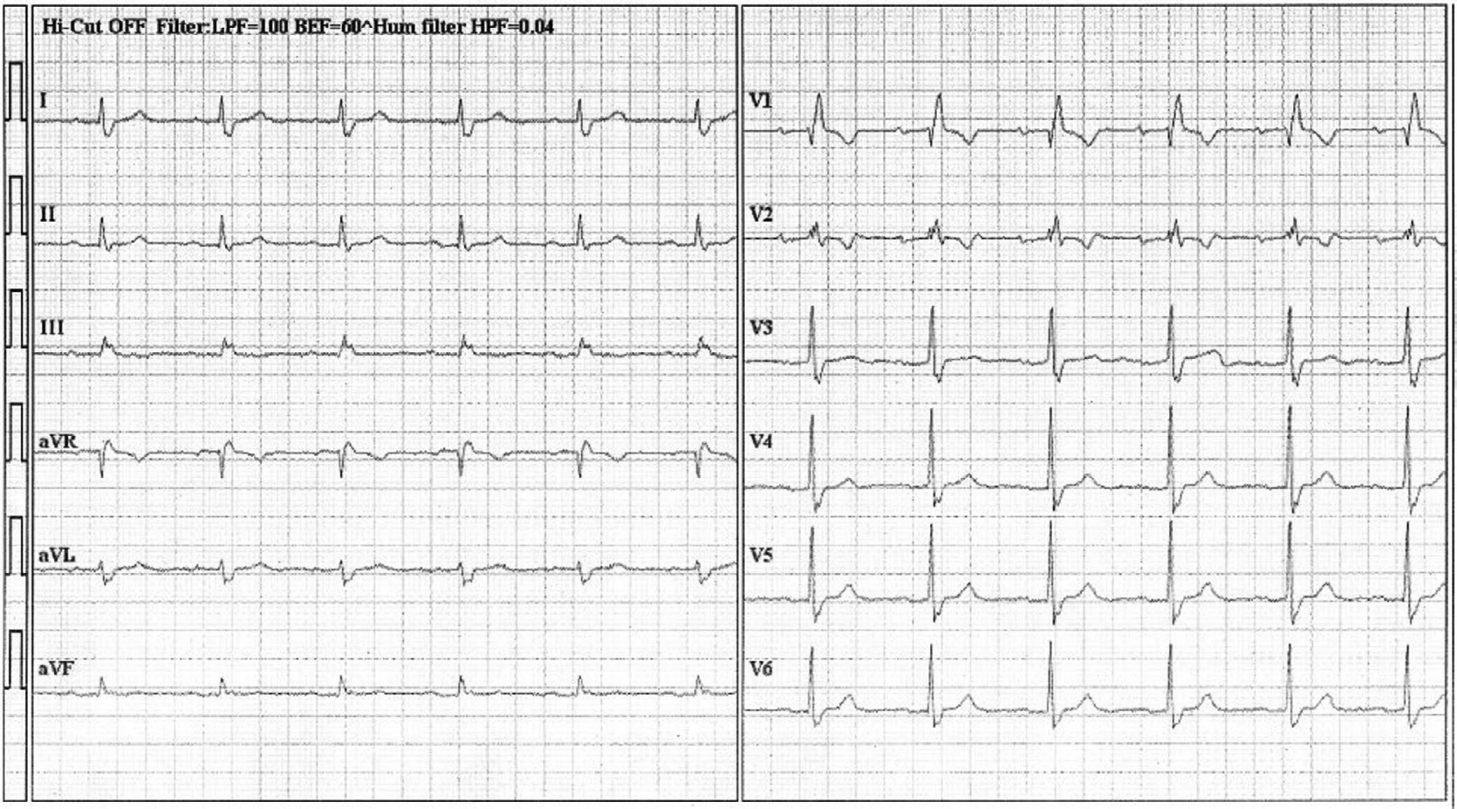
ECG at the time of ICU discharge. Bradycardia, QT prolongation, and Mobitz type II atrioventricular block are no longer observed

After approximately 3 days, bradycardia and hypersedation were ameliorated, and the Mobitz II type AV block disappeared. After discharge from the ICU, risperidone therapy was started, and the Mobitz II type AV block did not reappear (Figure 3). The patient's auditory hallucinations and agitation disappeared, and his extrapyramidal symptoms gradually improved. The patient had been undergoing rehabilitation for approximately 3 months. The patient was discharged on day 104 of hospitalization. Approximately two months later, he was admitted to our hospital and needed another round of antipsychotic medications. While receiving treatment, the Mobitz II type AV block did not recur. Currently, he reports to the psychiatric department of another hospital and receives medical treatment regularly. He also reports to the cardiovascular department of another hospital and undergoes routine ECG.

## 5 DISCUSSION

The patient received daily intramuscular injections of haloperidol and levomepromazine during a previous hospitalization, resulting in a temporary increase in antipsychotic dosage (measured in chlorpromazine [CP] equivalent), followed by an exacerbation of extrapyramidal symptoms, bradycardia, and QT prolongation. Antipsychotic dosage (CP equivalent) has been reported to be significantly associated with QT prolongation.^6^ Hypomagnesemia aggravates QT prolongation.^7^

Antipsychotic drugs reportedly block ion channels in the ventricles. Some antipsychotics suppress the rapidly activating delayed rectifier current potassium channels, and QT prolongation most commonly occurs secondary to potassium channel block.^8^ Our patient's QT prolongation improved after antipsychotic medication discontinuation and magnesium level correction. A relatively mild ion channel gene abnormality (gene polymorphism) in the myocardial stimulation conduction system has been thought to be involved in the development of secondary QT prolongation syndrome.^9^

Here, although QT prolongation improved, bradycardia and extrapyramidal symptoms persisted, and an unexpected Mobitz II type AV block was subsequently noted. We consulted cardiologists when treating our patient in the ICU, considering the indication for transcutaneous pacing. Patients who died from marked T‐wave abnormalities followed by complete AV block after receiving high‐dose thioridazine have been reported.^10^

Mobitz II type AV block usually shows a chronic course^1^, ^4^ and is an indication for pacemaker implantation. However, our patient's Mobitz II type AV block, which was induced by regular antipsychotic use, disappeared without recurrence after approximately 3 days. Reversible Mobitz II type AV block caused by antipsychotic drugs has rarely been reported, and the underlying mechanism is unknown.

We suspect that our patient's AV block occurred due to the antipsychotic medications’ indirect action on the autonomic nervous system, direct action on the impulse conduction system (of the heart), or both. Mobitz type II AV block can occur after clozapine administration,^11^ and there is a report of the development of Mobitz type II AV block associated with cocaine use that normalized on day 2 of hospitalization.^12^ Cocaine inhibits sodium channels and decreases sinoatrial node automaticity and AV node conduction. The slow attenuation of the pharmacological effects of the antipsychotics resulted in symptom improvement and reversible resolution of the Mobitz type II AV block approximately 72 h after transfer to our hospital. Therefore, the pharmacological effects of typical antipsychotics may be the main cause of reversible Mobitz type II AV block.

As the patient was extremely agitated, the haloperidol and levomepromazine doses were increased. However, since the elderly are at particular risk of adverse effects associated with antipsychotics, including mortality, dosage recommendations in are significantly more conservative than those for younger patients, and the medication should be titrated to the minimum effective dose.^13^ If antipsychotics cannot solely control agitation in the elderly, a combination of benzodiazepines, such as diazepam and midazolam, is preferred.^14^

In conclusion, ECG monitoring is useful for the early detection of drug‐induced arrhythmia when increasing the dose of antipsychotic drugs. The National Institute for Health and Care Excellence (NICE) guidelines recommend annual physical health monitoring for patients with schizophrenia on antipsychotic medication.^15^ This includes the monitoring of weight, blood pressure, fasting blood pressure, glycated hemoglobin, in addition to an ECG. Mobitz type II AV block should be considered in patients presenting with hypotension and bradycardia. Approximately 10% of sudden cardiac deaths in patients with schizophrenia occur from unknown causes.^16^ Mobitz type II AV blocks may be the cause of death in some of these cases.

## CLINICAL TRIAL REGISTRATION

Not Applicable.

## CONFLICT OF INTEREST

The authors declare no conflicts of interest.

## AUTHOR CONTRIBUTIONS

HN conceived and designed the study, analyzed and acquired the data, participated in drafting the manuscript; RT conceived and designed the study, participated in drafting the manuscript; HM analyzed and acquired the data; JK analyzed and acquired the data; KN‐N conceived and designed the study, participated in drafting the manuscript; YI conceived and designed the study, participated in drafting the manuscript; All authors read and approved the final manuscript. All authors agree to be accountable for all aspects of the work.

## ETHICS APPROVAL

Not applicable.

## CONSENT

Written informed consent was obtained from the patient to publish this report in accordance with the journal's patient consent policy.

## PERMISSION TO REPRODUCE MATERIAL FROM OTHER SOURCES

Not applicable.

## Data Availability

The datasets generated during and/or analyzed during the current study are not publicly available, but are available from the corresponding author on reasonable request.
